# POPULATION TRENDS IN HEALTHCARE USE BY MEXICAN ADULTS AGED 60 AND OLDER WITH AND WITHOUT COGNITIVE IMPAIRMENT

**DOI:** 10.1093/geroni/igac059.559

**Published:** 2022-12-20

**Authors:** Brian Downer, Jose Eduardo Cabrero Castro, Rebeca Wong

**Affiliations:** University of Texas Medical Branch, Galveston, Texas, United States; University of Texas Medical Branch, Galveston, Texas, United States; University of Texas Medical Branch, Galveston, Texas, United States

## Abstract

Government policies that have greatly expanded health insurance coverage in Mexico have taken place in the context of rapid population aging and an increasing number of older adults living with cognitive impairment. We used data from the Mexican Health and Aging Study to investigate population-level trends in self-reported healthcare use by cognitive status in 2001, 2012, 2015, and 2018. Healthcare measures included having an outpatient procedure, any doctor visits, staying >1 nights in the hospital, and screenings for high cholesterol, diabetes, and hypertension. All outcomes were dichotomized as yes/no. The sample sizes included 6179 (2001), 8924 (2012), 9429 (2015), and 8916 (2018) participants aged 60 and older who completed a direct interview (total N=33,448). Participants with cognitive impairment were identified using five cognitive assessments (2001 n=1000; 2012 n=1273; 2015 n=1467; 2018 n=1372). Generalized estimating equations that adjusted for demographic characteristics and self-reported health conditions were used. The adjusted odds of having spent >1 night in the hospital, outpatient procedures, any doctor visits, and preventive screenings were significantly higher in 2012, 2015, and 2018 than in 2001 regardless of cognitive status. Overall, participants with cognitive impairment had significantly higher adjusted odds for >1 nights in the hospital (OR=1.31, 95% CI=1.20-1.42), but significantly lower odds for any doctor visits (OR=0.81, 95% CI=0.75-0.88), outpatient procedures (OR=0.70, 95% CI=0.57-0.85), and preventive screenings for high cholesterol (OR=0.75, 95% CI=-.70-0.81), diabetes (OR=0.78, 95% CI=0.72-0.85), and hypertension (OR=0.76, 95% CI=0.70-0.82). These results are important to understanding the healthcare needs of Mexico’s growing older adult population.

